# A new *Antipathozoanthus* species (Cnidaria, Hexacorallia, Zoantharia) from the northwest Pacific Ocean

**DOI:** 10.3897/zookeys.1040.62309

**Published:** 2021-05-28

**Authors:** Hiroki Kise, Masami Obuchi, James Davis Reimer

**Affiliations:** 1 Graduate School of Engineering and Science, University of the Ryukyus, 1 Senbaru, Nishihara, Okinawa 903-0213, Japan; 2 Geological Survey of Japan, National Institute of Advanced Industrial Science and Technology, AIST Tsukuba Central 7, 1-1-1 Higashi, Tsukuba, Ibaraki 305-8567, Japan; 3 Endo Shell Museum of Manazuru, 1175 Manazuru, Manazuru, Kanagawa 259-0201, Japan; 4 Tropical Biosphere Research Center, University of the Ryukyus. 1 Senbaru, Nishihara, Okinawa 903-0213, Japan

**Keywords:** molecular phylogeny, polychaete, Sagami Bay, symbiosis, zoantharians

## Abstract

A new species of zoantharian within the genus *Antipathozoanthus* is described based on specimens collected from the coast of mainland Japan, northwest Pacific Ocean. *Antipathozoanthustubus***sp. nov.** is characterized by its substrate (epibiotic on polychaete tube) and habitat (exposed rock). As well, the results of molecular phylogenetic analyses using concatenated multiple genetic markers also support the distinction between *A.tubus***sp. nov.** and its congenerics. *Antipathozoanthustubus***sp. nov.** is the first species of *Antipathozoanthus* species reported to be epibiotic on polychaete tubes, and is the second species in the genus that is not associated with antipatharians.

## Introduction

The order Zoantharia Rafinesque, 1815 (Cnidaria: Anthozoa) consists of primarily colonial hexacorallians that are commonly found in most marine environments, including extreme environments such as intertidal zones and methane cold seeps in the deep sea (Reimer et al. 2007; Sinniger et al. 2010). The number of studies in Japanese waters on these species have increased in recent decades and have played key roles in the systematic re-appraisal and revision of zoantharians around the globe (Reimer and Fujii 2017). Moreover, the number of overall diversity records of zoantharian species from Japan has increased since 2006 by the addition of more than 20 formally described species (see Reimer and Fujii 2017). In particular, zoantharians within the suborder Macrocnemina Haddon & Shackleton, 1891 have been well studied in Japan as most newly described species belong to this suborder, and the total number of macrocnemic zoantharian species continues to rise by reports of many possibly undescribed species in Japanese waters (e.g., Sinniger et al. 2010; Reimer et al. 2010, 2019).

*Antipathozoanthus* Sinniger, Reimer & Pawlowski, 2010 within the family Parazoanthidae Delage & Hérouard, 1901 is a genus that has been the subject of recent research in Japanese waters (Sinniger et al. 2010; Kise et al. 2017). This genus currently contains five species (Reimer and Sinniger 2021), with records from Madagascar (Sinniger et al. 2010), the Red Sea, Palau, Maldives, Japan (Reimer et al. 2014; Kise et al. 2017; Reimer et al. 2019) in the Indo-West Pacific, and Ecuador (Reimer and Fujii 2010; Bo et al. 2012; Jaramillo et al. 2018) in the eastern Pacific Ocean, as well as from St. Helena (Santos et al. 2019), Cape Verde, Principe Islands (Ocaña and Brito 2003; Ocaña et al. 2007; Sinniger et al. 2010), and Curaçao (Montenegro et al. 2020) in the Atlantic Ocean and the Caribbean. As the generic name indicates, *Antipathozoanthus* species generally utilize antipatharians (Hexacorallia: Antipatharia) as their obligate substrate (Sinniger et al. 2010). However, *A.obscurus*Kise et al., 2017 described from Okinawa, Japan, and the Red Sea, is not associated with any antipatharians and instead is found in cracks and caves on coral-reef substrates (Kise et al. 2017). Thus, substrate specificity to antipatharians within the genus *Antipathozoanthus* is not all-inclusive, unlike as originally theorized (Sinniger et al. 2010).

Recently, we collected two specimens in Japanese waters of an undescribed species belonging to the genus *Antipathozoanthus*, which were unexpectedly found as epibionts on an empty polychaete tube. Here, we formally describe this new species, *Antipathozoanthustubus* sp. nov., utilizing morphological and phylogenetic data. With this addition, the entire Japanese zoantharian fauna now comprises 37 recorded species, representing 16 of the 28 currently-recognized genera across nine families (see also Reimer and Fujii 2017; Kise et al. 2017, 2018, 2019; Reimer et al. 2019).

## Materials and methods

### Specimen collection

The examined specimens were collected in shallow waters of Sagami Bay, Kanagawa, Japan on 2019 and 2020, by SCUBA (Table 1). Specimen images were taken in situ for gross external morphological observation.

**Table 1.** Information of specimens examined in this study.

**Familiy**	**Species**	**Voucher number**	**Locality**	**Coordinates**	**Date**	**Depth**	**Collecter**	**GenBank accession numbers**					
								**18S–rDNA**	**ITS–rDNA**	**28S–rDNA**	**COI**	**12S–rDNA**	**16S–rDNA**
Parazoanthidae	*Antipathozoanthustubus* sp. nov.	NSMT–Co 1742	Iwa Beach, Sagami Bay, Kanagawa, Japan	35°09'36"N, 139°08'36"E	26 Jul 2019	13.6	M. Obuchi	MW652773	MW652765	MW652768	MW649812	MW652761	MW652770
		NSMT–Co 1743	Kotogahama, Sagami Bay, Kanagawa, Japan	35°08'48"N, 139°09'05"E	6 Jul 2020	14	M. Obuchi	–	–	–	–	–	–
	* A. hickmani *	CMNH–ZG 05883	Roca Onan, Pizon Island, Galapagos, Ecuador	0°35'27.2"S, 90°41'09.6"W	14 Mar 2007	27	A. Chiriboga	MW652771	MW652764	–	–	MW652759	MW652769
	* A. cavernus *	NSMT–Co 1604	Sakurajima, Kagoshima, Japan	31°35'23.5"N, 130°35.27.8"E	20 Sep 2015	21	JD. Reimer	–	MG384699	MW652766	MG384660	MW652763	MG384681
	* A. remengesaui *	NSMT–Co 1603	Blue Hole, Palau	7°8'29.4"N, 134°13'23.3"E	15 Sep 2014	23	JD. Reimer	MW652772	MG384703	MW652767	MG384649	MW652762	MG384673
	* A. obscurus *	NSMT–Co 1602	Cape Bise, Motobu, Okinawa–jima Island, Japan	26°42'34.4"N, 127°52'49.2"E	14 Aug 2014	5	JD. Reimer	MW652774	MG384691	–	MG384644	MW652760	MG384685

### DNA extraction, PCR amplification, and sequencing

We extracted genomic DNA from tissue of the holotype specimen preserved in 99.5% EtOH using a spin-column DNeasy Blood and Tissue Extraction Kit (Qiagen, Hilden, Germany) following the manufacturer’s protocol. PCR amplification using Hot Star Taq Plus Master Mix Kit (Qiagen, Hilden, Germany) was performed for each of COI (mitochondrial cytochrome oxidase subunit I), mt 12S-rDNA (mitochondrial 12S ribosomal DNA), mt 16S-rDNA (mitochondrial 16S ribosomal DNA), 18S-rDNA (nuclear 18S ribosomal DNA), ITS-rDNA (nuclear internal transcribed spacer region of ribosomal DNA), and 28S-rDNA (nuclear 28S ribosomal DNA) using published primers (Medlin et al. 1988; Folmer et al. 1994; Apakupakul 1999; Chen et al. 2002; Sinniger et al. 2005; Swain 2009, 2010; Fujii and Reimer 2011). All PCR products were purified with 1 U of shrimp alkaline phosphatase (SAP) and 5 U of Exonuclease I (Takara Bio Inc., Shiga, Japan) at 37 °C for 40 min followed by 80 °C for 20 min. Cleaned PCR products were sequenced in both directions on an ABI 3730Xl Genetic Analyzer (Applied Biosystems, Thermofisher) at the Fasmac Co., Ltd., Kanagawa, Japan. Obtained sequences in this study were deposited in GenBank under accession numbers MW652759–MW652774.

### Molecular phylogenetic analyses

Forward and reverse sequences were assembled and edited in Geneious v10.2.3 (Kearse et al. 2012). Multiple alignments for each marker were performed with previously published Parazoanthidae sequences obtained from GenBank (Suppl. material 1: Table S1) using MAFFT ver. 7.110 (Katoh and Standley 2013) with the auto algorithm under default parameters for all genetic markers. In this study, sequences of two selected taxa within the zoantharian genus *Epizoanthus* were used as outgroups. We obtained a dataset of 549 bp for 34 sequences of COI, 757 bp for 22 sequences of mt 12S-rDNA, 772 bp for 40 sequences of mt 16S-rDNA, 1756 bp for 23 sequences of 18S-rDNA, and 902 bp for 33 sequences of ITS-rDNA, 936 bp for 16 sequences of 28S-rDNA. These alignments were subsequently concatenated to obtain a final dataset of 5672 bp for 40 OTUs. All aligned datasets are available from the first author and at treebase.org (ID: S27965).

Phylogenetic analyses were performed on the concatenated dataset using Maximum likelihood (ML) and Bayesian inference (BI). ModelTest-NG v0.1.6 (Darriba et al. 2019) under the Akaike information criterion was used to select the best fitting model for each molecular marker, independently for ML and BI. The best selected models for ML and BI analyses were HKY+G for COI, GTR+I+G for mt 12S-rDNA, SYM+I+G for mt 16S-rDNA, HKY+I+G for 18S-rDNA, TPM3uf+I+G (BI: HKY+I+G) for ITS-rDNA, and GTR+I+G for 28S-rDNA. Independent phylogenetic analyses were performed using model partition per each region in RAxML-NG v0.9.0 (Kozlov et al. 2019) for ML, and MrBayes v3.2.6 (Ronquist and Huelsenbeck 2003) for BI. RAxML-NG was configured to use 12345 initial seeds, search for the best tree among 100 preliminary parsimony trees, branch length was scaled and automatically optimized per partition, and model parameters were also optimized. MrBayes was configured following the models and parameters as indicated by ModelTest-NG, 4 MCMC heated chains were run for 5,000,000 generations with a temperature for the heated chain of 0.2. Chains were sampled every 200 generations. Burn-in was set to 1,250,000 generations at which point the average standard deviation of split frequency (ASDOSF) was steadily below 0.01.

### Morphological observations

Morphological data were collected from whole, dissected, and serial sections of the preserved specimens. Histological sections of 10–15 mm thickness were made using a RM-2125 RTS microtome (Leica, Germany) and were stained with hematoxylin and eosin after decalcification with a Morse solution for 48 h (1:1 vol; 20% citric acid: 50% formic acid). Classification of marginal muscle shapes followed Swain et al. (2015). Cnidae analyses were conducted using undischarged nematocysts from tentacles, column, actinopharynx, and mesenterial filaments of two polyps of holotype specimen under a Nikon Eclipse80i stereomicroscope (Nikon, Tokyo). Cnidae sizes were measured using ImageJ ver. 1.45 (Rasband, 2012). Cnidae classification generally followed England (1991) and Ryland and Lancaster (2004).

### Abbreviations

**NSMT** National Science Museum, Tsukuba, Ibaraki, Japan;

**CMNH** Coastal Branch of Natural History Museum and Institute, Chiba, Japan.

## Results

### Taxonomic description

#### Order Zoantharia Rafinesque, 1815

**Suborder Macrocnemina Haddon & Shackleton, 1891**

**Family Parazoanthidae Delage & Hérouard, 1901**

##### Genus Antipathozoanthus

Taxon classificationAnimaliaZoanthariaParazoanthidae

Sinniger, Reimer & Pawlowski, 2010

8E96ED0E-0F03-522E-A898-0079E9DF1A6E

###### Diagnosis

(revised from Sinniger et al. 2010; Swain and Swain 2014, 2015; Kise et al. 2017; additions in **bold**). Macrocnemic zoantharians with cteniform endodermal muscle or endo-meso transitional sphincter muscle. Encrustations of the column to the outer mesoglea. No mesogleal canals or encircling sinus. Tentacles at least 26 in number. Substrate consists of antipatharians, **external surfaces of parchment-like tubes of polychaetes**, or calcium carbonate (coral reef).

###### Type species.

*Gerardiamacaronesicus* Ocaña & Brito, 2003, by original designation.

###### Remarks.

We herein modify the diagnosis of *Antipathozoanthus*, as *A.tubus* sp. nov. is clearly located within the clade of *Antipathozoanthus* with very high support in our molecular phylogenetic analyses. Skeletal secretion as has been reported in *A.macaronesicus* (Ocaña & Brito, 2003) was not found in any other *Antipathozoanthus* species, including *A.tubus* sp. nov.

##### Antipathozoanthus tubus sp. nov.

Taxon classificationAnimaliaZoanthariaParazoanthidae

AD1D7441-F7A2-5799-A044-96BA84C9D908

http://zoobank.org/70CBDCBE-87C2-4A84-AF9D-D4841A082CEC

Figures 1 , 2 , 3

###### Material examined.

***Holotype*.**NSMT-Co 1742, collected from Iwa Beach, Sagami Bay, Kanagawa, Japan (35°09'36"N, 139°08'36"E) at a depth of 14 m by M. Obuchi, 26 July 2019, divided in two pieces, one portion fixed in 99.5% EtOH and the other in 5–10% saltwater formalin. ***Paratype*.**NSMT-Co 1743, collected from Kotogahama, Sagami Bay, Kanagawa, Japan (35°08'48"N, 139°09'05"E) at a depth of 14 m by M. Obuchi, 6 June 2020, divided in two pieces, one portion fixed in 99.5% EtOH and the other in 70% EtOH.

**Figure 1.**
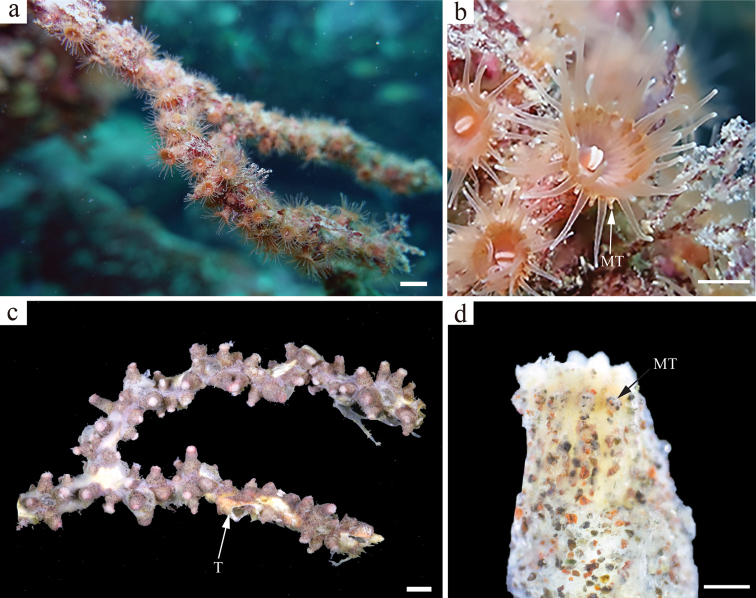
Images of external morphology of *Antipathozoanthustubus* sp. nov. (holotype: NSMT-Co 1742) **a** colony on branched polychaete tubes in situ **b** close-up image of polyps in situ **c** colony on branched polychaete tubes in preserved condition **d** close-up image of closed polyp. Abbreviations: MT: marginal teeth, T: tube of polychaete. Scale bars: 10 mm (**a, c**), 5.0 mm (**b**), 0.5 mm (**d**).

###### Material examined for comparison.

*Antipathozoanthusobscurus*NSMT-Co1602 (holotype), collected from Cape Bise, Motobu, Okinawa-jima Island, Japan, by J.D. Reimer, 14 August 2014. *Antipathozoanthusremengesaui*NSMT-Co1603 (holotype), collected from Blue Hole, Palau, by J.D. Reimer, 15 September 2014. *Antipathozoanthuscavernus*NSMT-Co1604 (holotype), collected from Sakurajima, Kagoshima, Japan, by J.D. Reimer, 20 September 2015. *Antipathozoanthushickmani*CMNH-ZG-05883 (paratype), collected from Roca Onan, Pinzon Island, Galapagos, Ecuador, by A. Chiriboga, 14 March 2007.

###### Type locality.

Iwa Beach, Sagami Bay, Kanagawa, Japan

###### Description.

***External morphology*.** Colonial zoantharian, with cylindrical polyps connected by well-developed dark red colored coenenchyme (Fig. 1a). External branched tube of dead polychaete mostly covered by coenenchyme. Scapus of column dark red in situ, dark brown in preserved specimens. Capitulum of column orange in situ, dark violet in preserved specimens. Column and coenenchyme heavily encrusted with visible sand and silica particles in ectodermal tissue to outer mesoglea (Fig. 1c, d). Preserved, contracted polyps 2.0–6.0 mm in height, 1.0–3.0 mm in diameter. In situ, opened polyps approximately < 8.0 mm in height, < 10 mm in diameter. Oral disk 5.0–8.0 mm in diameter, orange to light orange in coloration. Number of oral furrows the same as the number of tentacles, and cream white circular protrusion in central oral disk bears slit-like mouth aligned with directives. Tentacles arranged in two rows (15–17 inner endocoelic tentacles and 15–17 outer exocoelic tentacles), as long as the expanded oral disk diameter. Number of tentacles 30–34, transparent in coloration. 15–17 marginal teeth present under inner endocoelic tentacles (Fig. 1b). Tips of tentacles usually cream in coloration. Capitular ridges indiscernible.

**Figure 2.**
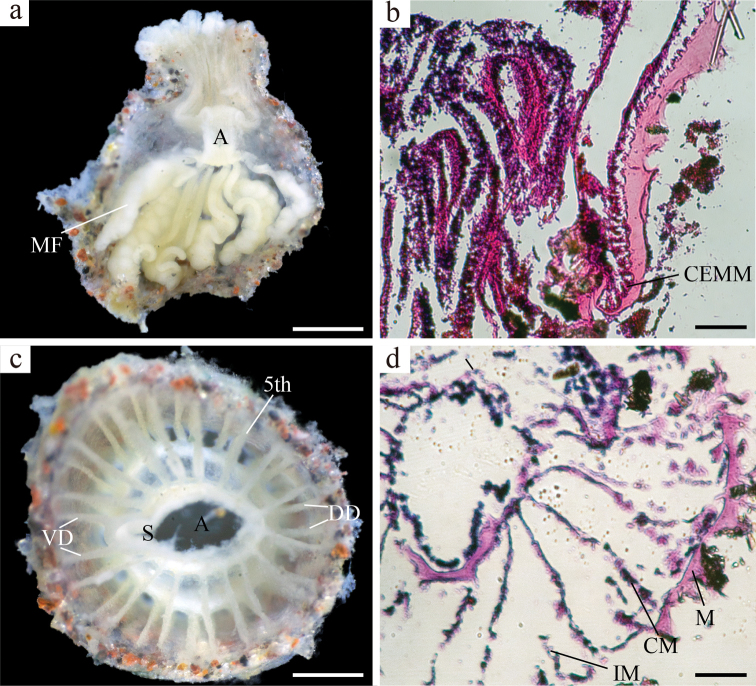
Image of internal morphology of *Antipathozoanthustubus* sp. nov. (holotype: NSMT-Co 1742) **a** longitudinal section of polyp **b** closed-up image of cteniform endodermal marginal muscle **c, d** cross-section of polyp. Abbreviations: A: actinopharynx, MF: mesenterial filament, CEMM: cteniform endodermal marginal muscle, DD: dorsal directives, VD: ventral directives, S: siphonoglyph, 5^th^: 5^th^ mesentery from dorsal directives, M: mesoglea, CM: complete mesentery, IM: incomplete mesentery. Scale bars: 0.5 mm (**a, c**), 0.1 mm (**b, d**).

***Internal morphology*.** Azooxanthellete. Mesentery number 30–34, complete 15–17, incomplete 15–17. Mesenteries in macrocnemic arrangement (Fig. 2c). Mesoglea thickness 0.01–0.10 mm, and thicker than ectoderm. Developed siphonoglyph distinct and U-shaped. Mesenterial filaments present (Fig. 2a). Endodermal marginal muscle, short comb-like mesogleal pleats supporting the entire length of the marginal muscle (cteniform endodermal marginal muscle: Fig. 2b). Basal canals of mesenteries absent (Fig. 2d). Additionally, possible gametes observed in several longitudinal sections.

**Figure 3.**
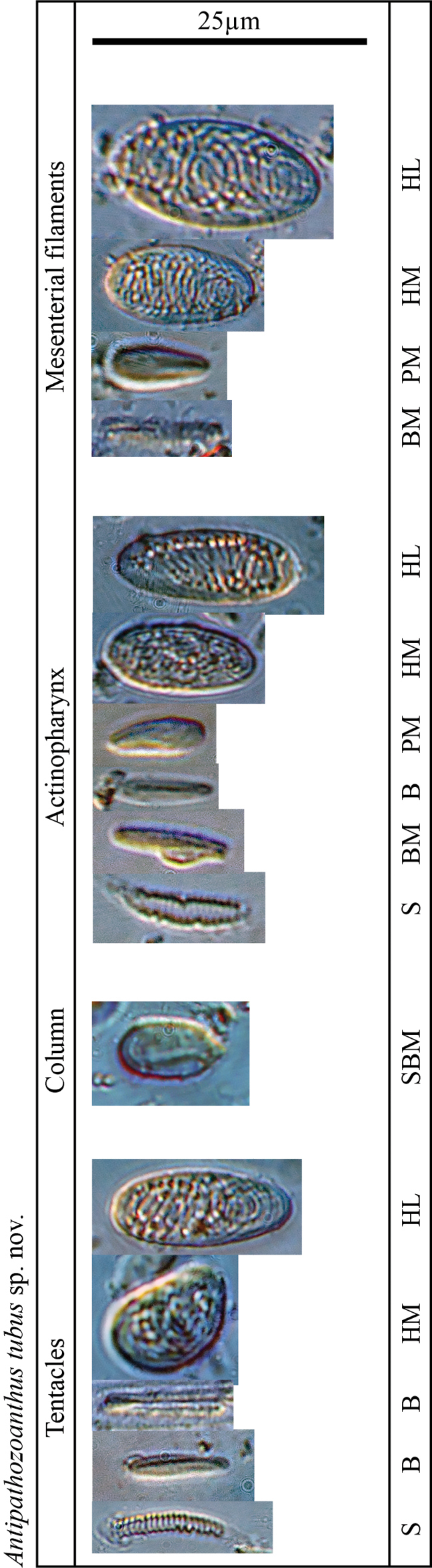
Cnidae in the tentacles, column, actinopharynx, and mesenterial filaments of holotype of *Antipathozoanthustubus* sp. nov. Abbreviations: HL: holotrich large, HM: holotrich medium, B: basitrichs, BM: microbasic *b*-mastigophores, SBM: special microbasic *b*-mastigophores, PM: microbasic *p*-mastigophores, S: spriocysts.

***Cnidae*.** Basitrichs and microbasic *b*-mastigophores, microbasic *p*-mastigophores, holotrichs, and spirocysts (Fig. 3, Table 2).

**Table 2.** Cnidae types and sizes observed in *Antipathozoanthustubus* sp. nov. Frequency: relative abundance of cnidae type in decreasing order; numerous, common, occasional, rare (n = number of cnidae).

**Tissue**	**Type of cnidae**	***Antipathozoanthustubus* sp. nov.**				
		**Length**	**Width**	**Mean±SD**	**Frequency**	**n**
		**(min-max)**	**(min-max)**	**(Length × Width)**		
Tentacles	Spirocysts	8.0–19.0	1.0–4.0	15.6±2.0 × 2.1±0.5	Numerous	325
	Bastrichs	7.0–16.0	1.0–4.0	10.4±1.5 × 2.0±0.7	Numerous	37
	Holotrichs medium	12.0–19.0	7.0–8.0	17.8±2.6 × 7.6±0.5	Occasional	6
	Holotrichs large	20.0–22.0	8.0–11.0	20.7±0.6 × 9.4±0.8	Occasional	10
Column	Special microbasic b-mastigophores	12.0	6.0	–	Rare	1
Actinopharynx	Spirocysts	10.0–16.0	1.0–3.0	12.7±1.6 × 2.4±0.7	Occasional	9
	Bastrichs	11.0–15.0	2.0–3.0	12.5±1.0 × 2.3±0.4	Numerous	37
	Microbasic b-mastigophores	8.0–15.0	2.0–3.0	10.0±1.9 × 2.6±0.5	Rare	5
	Microbasic p-mastigophores	9.0–11.0	3.0	10.0±0.8 × 3±0	Rare	3
	Holotrichs medium	16.0–19.0	5.0–8.0	18.3±0.1 × 6.8±0.1	Rare	4
	Holotrichs large	20.0–22.0	8.0–10.0	20.7±0.9 × 9.0±0.7	Occasional	15
Mesenterial filaments	Microbasic b-mastigophores	10.0–14.0	2.0–3.0	12.2±1.8 × 2.5±0.5	Rare	4
	Microbasic p-mastigophores	8.0–16.0	2.0–4.0	10.1±0.2 × 3.2±0.6	Numerous	60
	Holotrichs medium	12.0–19.0	5.0–10.0	17.8±1.9 × 9.3±1.2	Common	23
	Holotrichs large	20.0–25.0	10.0–12.0	21.1±1.2 × 10.7±0.5	Numerous	36

###### Habitat and distribution.

Northwestern Pacific Ocean: Sagami Bay, Kanagawa, Japan at depths < 14 m.

###### Associated host.

We could not identify host polychaete species as there were no polychaetes in the tubes. However, the tubes that *Antipathozoanthustubus* sp. nov. was attached to may belong to species within the genus *Eunice*, as polychaete species that build parchment-like branched tubes have been reported from this genus (e.g., Díaz-Díaz et al. 2020).

###### Molecular phylogeny.

**Figure 4.**
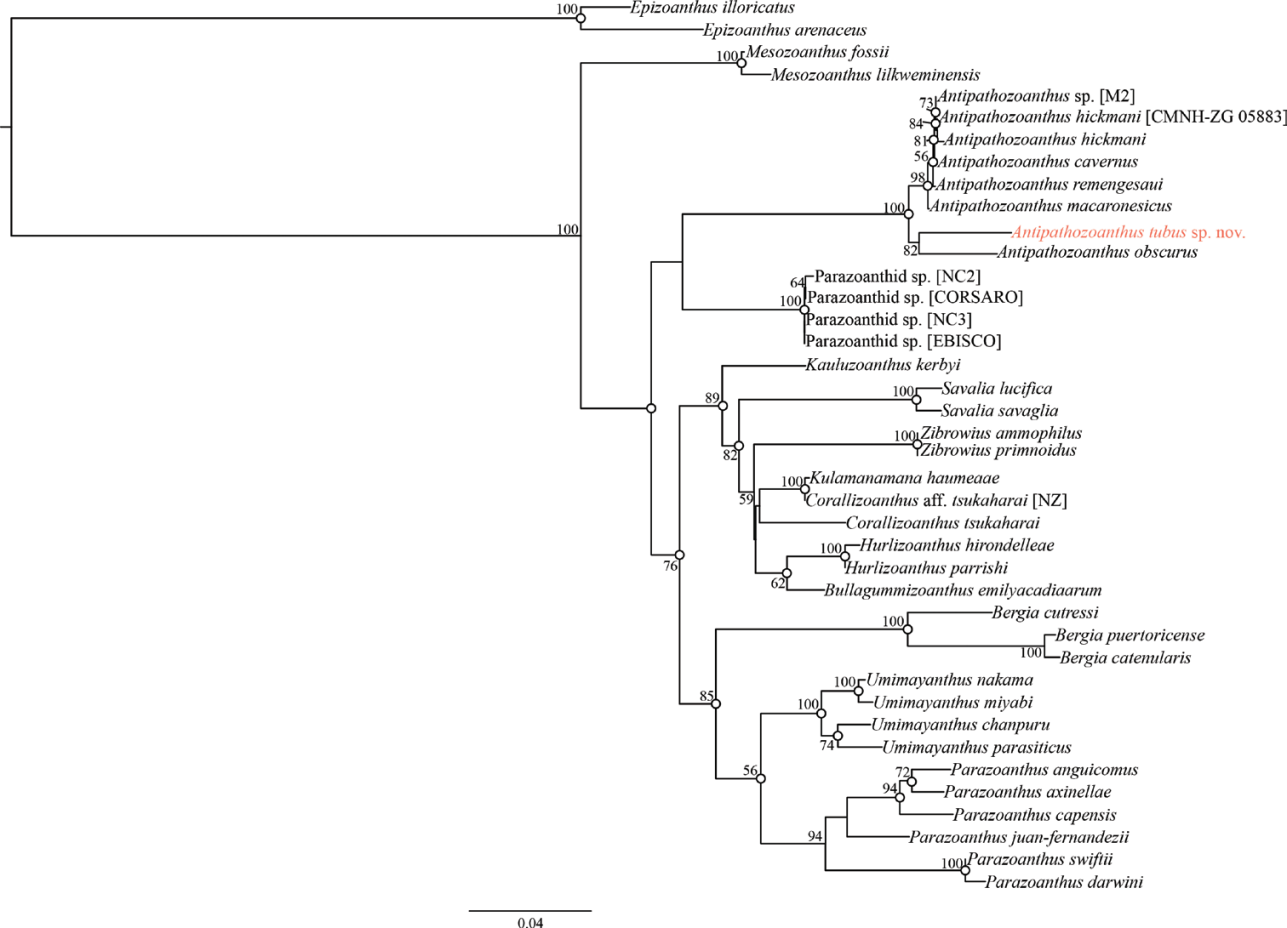
Maximum likelihood tree based on combined dataset of COI, mt 12S-rDNA, mt 16S-rDNA, 18S-rDNA, ITS-rDNA, and 28S-rDNA sequences. Number at nodes represent ML bootstrap values (> 50% are shown). White circles on nodes indicate high support of Bayesian posterior probabilities (>0.95).

Both ML and BI phylogenetic analyses showed similar topologies as indicated in Fig. 4. The genus *Antipathozoanthus* appeared as a monophyletic clade located within the family Parazoanthidae with strong nodal support (ML=100%, BI=1) and was close to a Parazoanthidae clade containing species associated with stalked hexactinellid sponges. Within *Antipathozoanthus*, two subclades were formed; one subclade consisted of the antipatharian-associated species *A.macaronesicus*, *A.hickmani*, *A.remengesaui*, and *A.cavernus* (ML = 100%, BI = 0.97), and the other subclade consisted of *A.tubus* sp. nov. and *A.obscurus* (ML = 82%, BI = 0.92). Genetic distances in COI, 16S-rDNA, and ITS-rDNA sequences between *A.tubus* sp. nov. and other *Antipathozoanthus* species were 0.000 to 0.009, 0.002 to 0.010, and 0.010 to 0.128, respectively. As well, *A.tubus* sp. nov. and other *Antipathozoanthus* species shared unique insertion/deletion patterns in 16S-rDNA sequences.

###### Remarks.

*Antipathozoanthustubus* sp. nov. can be easily distinguished from *A.remengesaui*Kise et al., 2017, *A.macaronesicus* (Ocaña & Brito, 2003), and *A.hickmani* Reimer & Fujii, 2010 by the number of tentacles as well as different coloration; *Antipathozoanthusremengesaui*, *A.macaronesicus*, and *A.hickmani* have up to 42 tentacles (Ocaña and Brito 2003; Reimer and Fujii 2010; Kise et al. 2017), while *A.tubus* sp. nov. has fewer tentacles (30–34). The dark red colored polyps and coenenchyme of *A.tubus* sp. nov. are not found in these other three *Antipathozoanthus* species. In addition, *A.tubus* sp. nov. differs from *A.cavernus*Kise et al., 2017 with regards to polyp coloration (*A.cavernus* has orange or light orange polyps: Kise et al. 2017). Although *A.tubus* sp. nov. and *A.obscurus*Kise et al., 2017 are phylogenetically close, their COI, 16S-rDNA, and ITS-rDNA sequences are all unique (genetic distances in COI, 16S-rDNA and ITS-rDNA sequences between *A.tubus* sp. nov. and *A.obscurus* were 0.009, 0.03, and 0.12, respectively). As well, these two species can be separated by coloniality; polyps of *A.obscurus* are connected by a stolon forming a mesh network (Kise et al. 2017), while polyps of *A.tubus* sp. nov. are connected by a well-developed coenenchyme. Furthermore, *A.macaronesicus*, *A.remengesaui*, *A.cavernus*, *A.hickmani*, and *A.obscurus* have holotrichs in their column (Ocaña and Brito 2003; Reimer and Fujii 2010; Kise et al. 2017), while holotrichs were not observed in the column of *A.tubus* sp. nov.

*Antipathozoanthus* is a circumglobally distributed genus, as species have reported from the Indian, Pacific, and Atlantic Oceans (Ocaña and Brito 2003; Sinniger et al. 2010; Reimer and Fujii 2010; Bo et al. 2012; Reimer et al. 2014; Kise et al. 2017), with members living from shallow waters (*A.obscurus* at 3 m depth; Kise et al. 2017) to mesophotic depths (153–169 m for *Antipathozoanthus* sp. sensu Reimer et al. 2019). The most distinctive attributes of *A.tubus* sp. nov. are its substrate and habitat. *Antipathozoanthusmacaronesicus*, *A.hickmani*, A.remengesaui, and *A.cavernus* are found on antipatharians within the families Antipathidae and Myriopathidae (Ocaña and Brito 2003; Reimer and Fujii 2010; Sinniger et al. 2010; Bo et al. 2012; Kise et al. 2017), while *A.obscurus* is directly attached to coral reef carbonate (Kise et al. 2017). On the other hand, *A.tubus* sp. nov. is the only species of the genus found to date on tubes of polychaetes. Four *Antipathozoanthus* species are known from low light environments; *A.macaronesicus*, *A.remengesaui*, and *A.cavernus* have been found in cave entrances, and *A.obscurus* is found in crevasses and/or coral reef caves (Ocaña and Brito 2003; Kise et al. 2017). On the other hand, the habitat of *A.tubus* sp. nov. is not a low-light environment, but the specimens were instead found on a polychaete tube attached to exposed rock.

Within Parazoanthidae, until now, *Isozoanthusaltisulcatus* Carlgren, 1939 is the only species described as living on the tubes of polychaetes. However, several morphological differences exist between *A.tubus* sp. nov. and *I.altisulcatus*. Capitular ridges are developed and conspicuous in *I.altisulcatus*, whereas they are indiscernible in *A.tubus* sp. nov. The marginal teeth on the capitulum found in *A.tubus* sp. nov. were not observed in *I.altisulcatus*. Although Carlgren (1939) did not describe the numbers of tentacles of *I.altisulcatus*, the numbers of mesenteries are 34–42 (Carlgren 1939). As numbers of tentacles are known to be equal to the number of mesenteries (Bourne 1900), the number of tentacles of *I.altisulcatus* is likely to be 34–42, which is greater than the number of tentacles of *A.tubus* sp. nov. (30–34).

Genetic distances of COI sequence between *A.tubus* sp. nov. and other *Antipathozoanthus* species can be considered as intra-generic differences based on previous comparisons of genetic distances (Sinniger et al. 2010). As well, *A.tubus* sp. nov. shared unique insertion/deletion patterns in 16S-rDNA sequences with other *Antipathozoanthus* species. Thus, we consider that *A.tubus* sp. nov. should belong to the genus *Antipathozoanthus* and does not warrant the erection of a new parazoanthid genus.

###### Etymology.

*Antipathozoanthustubus* sp. nov. is named from the Latin *tuba*, as this species is found on polychaete tubes. The Japanese name is ‘Iwa-tsuno-sunaginchaku’.

## Discussion

Japanese waters are composed of a wide variety of physical, geographical, and topographical environments due to the latitudinal extension of Japan spanning from the near-tropics of Okinawa to the near-subarctic Hokkaido, and also to the dynamic geology of the region, and thus, Japanese waters have high marine species diversity levels (Fujikura et al. 2010). At the same time, it is estimated that more than 70% of the marine taxa in this region remain undescribed (Fujikura et al. 2010). The order Zoantharia is one such taxon for which much work remains to be done. Although many zoantharian studies have been conducted in Japan, taxonomic studies are still biased by region; southern Japan including Kochi and the Ryukyu Archipelago have been focused on in comparison to other regions (Reimer and Fujii 2017). As a result, 16 species have been described based on type specimens collected from southern Japan (mainly from the Ryukyu Archipelago) (e.g., Irei et al. 2015). In other regions, historical taxonomic works have been conducted in Sagami Bay by Lwowsky (1913), Tischbierek (1929), and Carlgren (1934), with the description of three macrocnemic species; *Hydrozoanthusgracilis* Lwowsky, 1913, *Epizoanthuscnidosus* Tischbierek, 1929 (junior synonym of *Hydrozoanthusgracilis*), and *Epizoanthusramosus*, Carlgren 1934. As well, Hertwig (1882) reported the carcinoecium-forming *Epizoanthusparasiticus* (Verill, 1864) based on the specimens collected from the Sea of Enshu during the Challenger expedition. However, few taxonomic studies have been conducted in these regions since these past historical works, and for many other regions, almost no literature exists (e.g. the Sea of Japan). Thus, in order to understand species richness and the distribution patterns of zoantharians in Japan, further diversity studies with sampling efforts focused on understudied regions are required.

## Supplementary Material

XML Treatment for Antipathozoanthus

XML Treatment for Antipathozoanthus tubus

## Supplementary materials

Supplementary material 1 Table S1 Data type GenBank accession numbers Explanation note GenBank accession numbers used for phylogenetic analyses in this study. Newly obtained sequences indicated in bold. https://binary.pensoft.net/file/548330This dataset is made available under the Open Database License (http://opendatacommons.org/licenses/odbl/1.0/). The Open Database License (ODbL) is a license agreement intended to allow users to freely share, modify, and use this Dataset while maintaining this same freedom for others, provided that the original source and author(s) are credited.Hiroki Kise, Masami Obuchi, James Davis Reimer
